# Measuring health inequalities in Albania: a focus on the distribution of general practitioners

**DOI:** 10.1186/1478-4491-4-5

**Published:** 2006-02-21

**Authors:** Pavlos N Theodorakis, Georgios D Mantzavinis, Llukan Rrumbullaku, Christos Lionis, Erik Trell

**Affiliations:** 1 Clinic of Social and Family Medicine, School of Medicine, University of Crete, Heraklion, Crete, Greece; 2 State Mental Health Hospital of Chania, Chania, Crete, Greece; 3 Department of General Practice and Primary Health Care, Faculty of Health Sciences, University of Linköping, Linköping, Sweden; 4 Research Unit, Cretan Mental Health Services Coordination Centre, Chania, Crete, Greece; 5 Department of Family Medicine, Faculty of Medicine, University of Tirana, Tirana, Albania

## Abstract

**Background:**

The health workforce has a dynamically changing nature and the regular documentation of the distribution of health professionals is a persistent policy concern. The aim of the present study was to examine available human medical resources in primary care and identify possible inequalities regarding the distribution of general practitioners in Albania between 2000 and 2004.

**Methods:**

With census data, we investigated the degree of inequality by calculating relative inequality indices. We plotted the Lorenz curves and calculated the Gini, Atkinson and Robin Hood indices and decile ratios, both before and after adjusting for mortality and consultation rates.

**Results:**

The Gini index for the distribution of general practitioners in 2000 was 0.154. After adjusting for mortality it was 0.126, while after adjusting for consultation rates it was 0.288. The Robin Hood index for 2000 was 11.2%, which corresponds to 173 general practitioners who should be relocated in order to achieve equality. The corresponding figure after adjusting for mortality was 9.2% (142 general practitioners), while after adjusting for consultation rates the number was 20.6% (315). These figures changed to 6.3% (100), 6.3% (115) and 19.8% (315) in 2004.

**Conclusion:**

There was a declining trend in the inequality of distribution of general practitioners in Albania between 2000 and 2004. The trend in inequality was apparent irrespective of the relative inequality indicator used. The level of inequality varied depending on the adjustment method used. Reallocation strategies for general practitioners in Albania could be the key in alleviating the inequalities in primary care workforce distribution.

## Background

For over 40 years Albania had a Stalinist economy, in which the means of production came under the principle of controlled planning and state ownership [1,2]. During this period, the health sector in Albania was not considered to be a productive element of economy and was therefore given less importance in terms of finance and human resources development. Today the health system in Albania, as well as the country as a whole, is in a state of continuous transition, at a time when it still feels weak and exhausted by the previous regime and the slowness of its reform [3,4].

As Albania has moved during the last decade towards a national health care system that emphasizes the development of primary care [1,2], there is a debate on how to ensure that quality of care is guaranteed to all Albanians [1-3]. Quality can be seen as the measure by which satisfactory responses are provided to meet people's health needs and problems. Quality and equity have been considered together as the key concepts of the WHO strategy "Towards Unity for Health". Both concepts are closely linked with the health workforce, and especially with those serving in primary health care [5].

The health workforce has a dynamically changing nature and the regular documentation of the distribution of health professionals is a persistent policy concern [6-8]. Thus, it is of great interest to study available human medical resources in primary care and identify possible inequalities regarding the distribution of general practitioners in Albania. In Albania, general practitioners are physicians working in primary care as patients' first point of contact with organized medical services. Postgraduate training in family medicine was introduced in Albania only in 1997 [9].

Most doctors holding general practitioner posts are not specialized; they are general physicians. The remaining general practitioner workforce consists of specialized physicians (family doctors) who also hold general practice posts. It is the responsibility of the regional branches of the Health Insurance Institute to assess the changing needs of general practitioner posts in each district. District Public Health Directorates are then responsible for recruiting or relocation of general practitioners.

In the present study we calculated the level of inequality in the distribution of general practitioners and the number of general practitioners who must be relocated in order to achieve an equitable distribution, adjusting for population health needs.

## Methods

### Setting

Data on general practitioners by district during 2000–2004 were obtained from the Health Insurance Institute of Albania. All the general practitioners were clinically active, full-time state employees of the Health Insurance Institute of Albania. The study included the average annual population in each of the 36 districts of Albania as reflected by the Albanian National Institute of Statistics for each studied year [10,11].

### Variables

We calculated the general practitioners per 10 000 population ratio (GPPR) and the need-adjusted index (NAI). We used two variables as indicators of population health need: crude mortality rate (CMR) per 10 000 population and consultation rates per population. The NAI was calculated by dividing the GPPR by CMR (NAIM) in the first case, as previously suggested [12,13], and consultation rates in the second (NAIC). Data concerning mortality figures and consultation rates were obtained from the Albanian National Institute of Statistics [10,11].

### Analyses

In the present study we calculated the level of inequality in the distribution of general practitioners in Albania and the number of general practitioners who must be relocated in order to achieve an equitable distribution. For that purpose we used relative inequality indicators: we plotted the Lorenz curves [14,15] and calculated the Gini coefficient [14], decile ratio [6], Atkinson index [6] and Robin Hood index [16]. Inequality in all cases was estimated both before and after adjustment for population health need for the studied years. Inequality indices were calculated using the software "DAD" [17]. Graphs were plotted using Microsoft Excel software for Windows.

### Inequality indicators

#### Lorenz curve

The Lorenz curve compares the distribution of a specific variable with the uniform distribution that represents equality [14,15,18]. This equality distribution is represented by a diagonal line, and the greater the deviation of the Lorenz curve from this line, the greater the inequality. In order to safely compare two Lorenz curves, these curves should not cross. If the curves do not cross, then the one closest to the diagonal represents the least unequal distribution. The cumulative proportion of the population is generally shown on the X axis, and the cumulative proportion of the health variable on the Y axis. The greater the distance from the diagonal line, the greater the inequality.

#### Gini index

The Gini coefficient, one of the most commonly used indicators of inequality [6,7,14,15,18-21], is derived from the Lorenz curve. The Gini is calculated as the ratio of the area between the Lorenz curve and the 45° line, to the whole area below the 45° line. There are different methods to calculate the Gini index; in the present study we used the formula provided by M. Brown [14]:



*G*: Gini coefficient

*Yi*: Cumulative proportion of the health variable (GPs) in the *ith *district

*Xi*: Cumulative proportion of the population variable in the *ith *district

*k*: total number of districts.

#### Decile ratio

In order to calculate the decile ratio, districts are ranked by GPPR ratios. The top 10% from the top ratio is then divided by the 10% of the bottom [6].

#### Atkinson index

The Atkinson index is one of the few inequality measures that explicitly incorporate normative judgments about social welfare. The index is derived by calculating the equity-sensitive average studied variable (*Y*), which is defined as the level of per capita GPs that – if they provides services to everybody – would make total welfare exactly equal to the total welfare generated by the actual distribution of GPs. The index is given by the formula:



where *Y*_*i *_is the proportion of total number of GPs within the *i*th group, and *ε *is the inequality aversion parameter. The parameter *ε *reflects the strength of society's preference for equality, and can take values ranging from zero to infinity. When *ε *> 0 there is a social preference for equality, or in other words an aversion to inequality. As *ε *rises, society attaches more weight to transfers at the lower end of the distribution and less weight to transfers at the top. The more equal the distribution, the closer *Y*_*ε *_will be to *μ*, and the lower the value of the Atkinson index. For any given distribution of GPs, the value of *I *lies between 0 and 1.

#### Robin Hood index

The Robin Hood index is equivalent to the maximum vertical distance between the Lorenz curve and the line of equality. The value of the index approximates the share of the total number of GPs who must be transferred from districts above the ratio for the country to those below that figure to achieve equality in the distribution of GPs in all districts [16].

## Results

There was a variation in the distribution of GPs in Albania during 2000 and 2004. The GPPR for Albania was 4.5 during 2000, while it was 5.1 for 2004.

**Figure 1:**
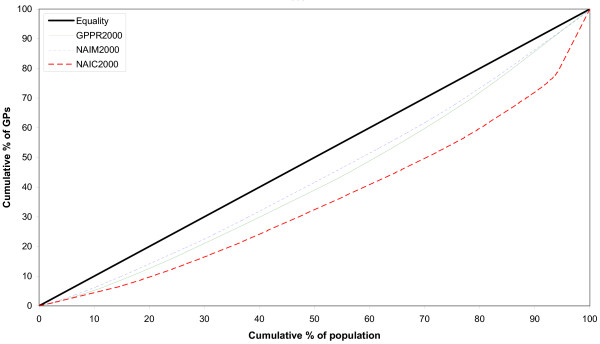
**Lorenz curves of the distribution of general practitioners in Albania for the year 2000**. GPPR = General Practitioners per Population Ratio, NAIM = Need Adjusted Index for Mortality, NAIC = Need Adjusted Index for Consultation rates.

**Figure 2:**
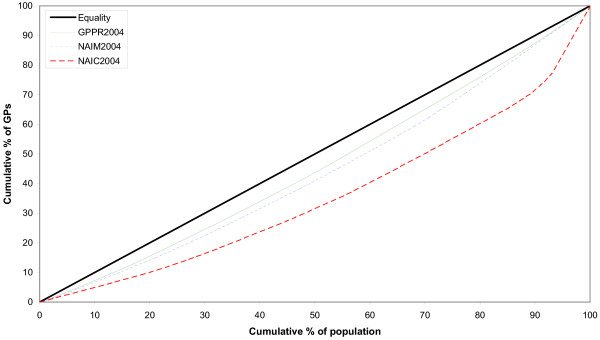
**Lorenz curves of the distribution of general practitioners in Albania for the year 2004**. GPPR = General Practitioners per Population Ratio, NAIM = Need Adjusted Index for Mortality, NAIC = Need Adjusted Index for Consultation rates.

Lorenz curves both before and after adjustment for population health needs are represented in Figs. 1 and 2. The curve corresponding to the adjustment for population health needs by mortality (NAIM) during 2000 is closer to the diagonal, compared to the curve representing the adjustment for population. On the contrary, the curve representing the adjustment for consultation rates (NAIC) was the furthest from the diagonal of equality (Fig. 1). During 2004, the NAIM Lorenz curve was closer to the diagonal, compared to the curve of GPPR. Still, the curve adjusted for consultation rates is further from the diagonal than all the other curves (Fig. 2).

The Gini index for GPPR was 0.154 (Table 1). After adjusting for mortality it was found to be 0.126, while after adjusting for consultation rates it was 0.288. The unadjusted decile ratio for the distribution of GPPR was 0.872. After adjusting for mortality it was 0.609, while after adjusting for consultation rates it was 0.958. The Atkinson index for GPPR was 0.0483, while for mortality and consultation rates it was 0.0265 and 0.1287, respectively.

**Table 1:** Relative inequality indicators of the distribution of general practitioners in Albania between 2000 and 2004

**Year**	**Gini index**			**Atkinson index**			**Decile ratio**			**Robin Hood index**		
	
	**GPPR**	**NAIM**	**NAIC**	**GPPR**	**NAIM**	**NAIC**	**GPPR**	**NAIM**	**NAIC**	**GPPR**	**NAIM**	**NAIC**
2000	0.154	0.124	0.288	0.0483	0.0265	0.1348	0.872	0.609	0.356	11.2	9.2	20.6
2001	0.126	0.154	0.275	0.0270	0.0391	0.1199	0.603	0.536	0.253	10.4	11.3	19.4
2002	0.107	0.142	0.263	0.0202	0.0340	0.1093	0.636	0.560	0.257	8.4	10.4	18.2
2003	0.092	0.098	0.308	0.0139	0.0163	0.1410	0.657	0.629	0.250	6.9	7.2	22.3
2004	0.086	0.123	0.287	0.0124	0.0238	0.1311	0.642	0.564	0.289	6.3	9.1	19.8

GPPR: General practitioners per population ratio

NAIM: Needs-adjusted index for mortality

NAIC: Needs-adjusted index for consultation rates

When the Robin Hood index for the year 2000 is taken under consideration, 173 general practitioners should be reallocated in order to achieve equality in their distribution. The corresponding figure after adjusting for mortality was 142 general practitioners, while after adjustment for consultation rates the number was 315. When the number of GPs for the year 2004 was taken under consideration, the Gini index was 0.086. The Robin Hood index was 6.3, indicating that 101 physicians had to be reallocated in order to achieve an equitable distribution for the whole country.

**Figure 3:**
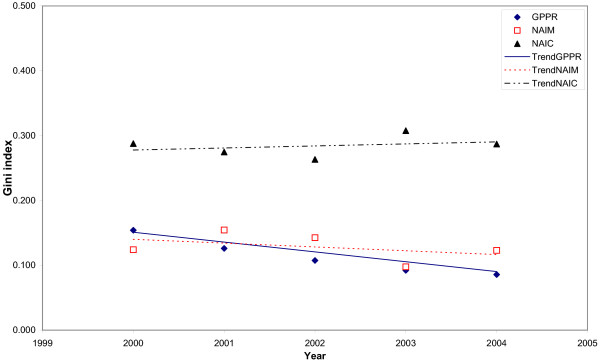
**Trends in the Gini index for the distribution of general practitioners in Albania between 2000 and 2004 with different adjustments**. Trends are represented by the linear trends in each studied year. GPPR = General Practitioners per Population Ratio, NAIM = Needs-Adjusted Index for Mortality, NAIC = Needs-Adjusted Index for Consultation rates.

**Figure 4:**
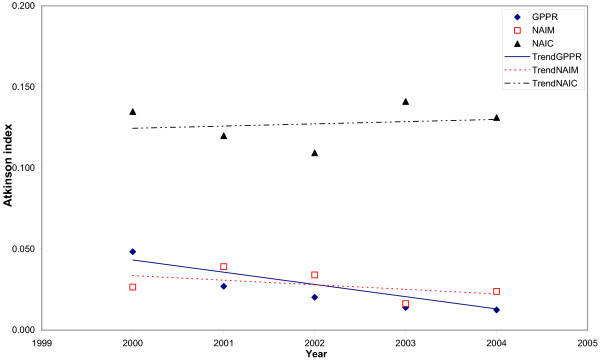
**Trends in the Atkinson index for the distribution of general practitioners in Albania between 2000 and 2004 with different adjustments**. Trends are represented by the linear trends in each studied year. GPPR = General Practitioners per Population Ratio, NAIM = Needs-Adjusted Index for Mortality, NAIC = Needs-Adjusted Index for Consultation rates.

**Figure 5:**
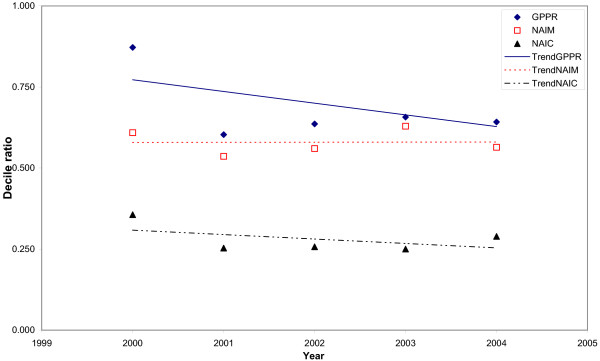
**Trends in the decile ratio for the distribution of general practitioners in Albania between 2000 and 2004 with different adjustments**. Trends are represented by the linear trends in each studied year. GPPR = General Practitioners per Population Ratio, NAIM = Needs-Adjusted Index for Mortality, NAIC = Needs-Adjusted Index for Consultation rates.

**Figure 6:**
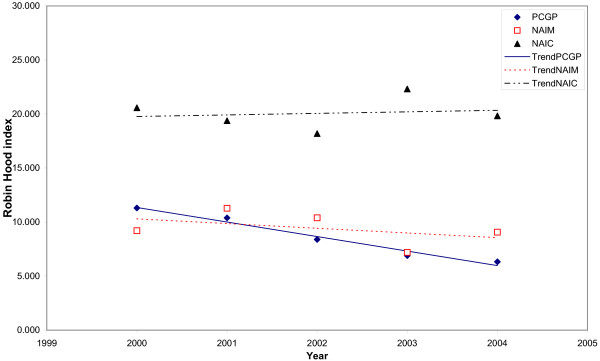
**Trends in the Robin Hood index for the distribution of general practitioners in Albania between 2000 and 2004 with different adjustments**. Trends are represented by the linear trends in each studied year. GPPR = General Practitioners per Population Ratio, NAIM = Needs-Adjusted Index for Mortality, NAIC = Needs-Adjusted Index for Consultation rates.

Trends in the inequalities with different inequality indicators and different adjustments are shown in Figs. 3, 4, 5 and 6. When the Gini index (Fig. 3), Atkison index (Fig. 4), decile ratio (Fig. 5) and Robin Hood index (Fig. 6) are used, there is a decreasing trend in terms of the GP per population ratio. The Gini, Atkinson and Robin Hood indices appear to have similar trends in all cases.

## Discussion and conclusion

### Influence of different need adjustments on inequality of GP supply

General practitioners are inequitably distributed in Albania. This becomes apparent when all the relative inequality indices are examined. In order to achieve an equal distribution of general practitioners in all districts of Albania by redistributing the existing human resources, more than one in 10 general practitioners should be relocated from relatively overserved to relatively underserved districts in 2000. After the adjustment for community health needs (mortality), the proportion of general practitioners that should be relocated in order to achieve an equitable distribution of the primary care medical workforce is less. The contrary was true, however, when consultation rates were used as population health needs adjustment. In this case, more – approximately two out of 10 – general practitioners needed to be relocated in order to meet population needs.

### International comparisons

Similar studies have been conducted in the past in other countries, such as the USA [19], UK [6,8], Sweden [7], Japan [20] and Thailand [22]. As previously discussed [21], we should be cautious when comparing inequalities in the distribution of the health workforce between different countries. This is because of the possible differences in health systems and health care provision, differences in geographical divisions and differences in the intracountry cross-boundary flows. Despite all this, we believe that relative inequality could also be used for comparison purposes, either in terms of trends within the same country or for intercountry comparison.

Horev et al. [19], using states as the unit of measure of Gini coefficient, concluded that overall inequality in the distribution of physicians in the USA was rising despite the increase in the ratio of physicians per population over the study period. In their study of the trends in the inequalities in the distribution of GPs in the UK, Hann and Gravelle [8] reported that the inequality in the distribution of GPs, as measured by the Gini coefficient, increased from the mid-1980s to 2003. They concluded that even the increase in the number of GPs did not reduce the maldistribution.

That increasing the supply of human resources does not necessarily lead to decline of the maldistribution has also been reported in a study by Kobayashi and Takaki in Japan [20]. They reported that even though there was a significant increase in the supply of physicians throughout the country, they were still unevenly distributed because they preferred municipalities with higher population density. In a study by Nishiura et al. [22] it was demonstrated that there are inequalities in the distribution of physicians (Gini index = 0.433) by province. As far as Sweden is concerned [7], in 1986 the Gini coefficient for the distribution of GPs was 0.086, while in 2001 it was 0.071.

### Methodological implications

It is apparent that the level of inequality in the distribution of GPs in a given year depends on the needs-adjustment method used. In contrast, trends remain unchanged regardless of the relative inequality indicator used. There are, however, some methodological issues that should be taken under consideration. There is a concern as to whether CMR represents an appropriate indicator for measuring a population's need for primary care provision [6]. For that reason we also adjusted for consultation rates, which possibly reflect population need more accurately. Other possible indicators would be measures of self-reported health status or limiting long-term illness, both reflecting more directly than CMR the need for primary care services [6]. Furthermore, analysis and comparison with previous years was not possible, because data on human health resources were destroyed during the 1997 civil war.

### Policy implications

Inequality in the distribution of GPs shows a decreasing trend throughout the studied period. This decrease does not seem to have been a result of health policy planning changes, because there is no report of any health policy measure during these years that could have had such an impact. It seems, though, that the underlying reason for this decrease is twofold: first, there is an increase in the overall number of GPs working in Albania (1531 in 2000; 1579 in 2004). This fact alone does not necessarily lead to a more equal distribution in terms of relative inequality measures [6]. It was, however, combined with changes in the number of GPs within each district. Moreover, there has been a change in the population of each district, explained mainly by the migration process towards developed countries, which has been occurring in waves during the last decade.

It seems that without any specific policy change towards the health workforce, a more equal distribution has been achieved through the study period. But this could easily be reversed without an effective policy. When consultation rates are adjusted for, it seems that trends remain stable throughout the study period, and inequalities are high regardless of the indicator used. Effective policies are needed in order to achieve a stable, equitable distribution of the health workforce and increase the opportunity for equal access to primary care services for the Albanian population.

Policy-makers should focus on three main issues that interact with each other, in order to achieve and maintain equality in the distribution of the health workforce [6]. First, increase in the provision of GPs seems to be imperative if improved primary care services provision is to be achieved. Increase in the overall provision of GPs will not necessarily lead to a more equal or equitable distribution of GPs, although all districts will be supplied with more physicians. Second, incentives at the local level could play an important role in health workforce provision. These incentives could be both financial and educational, focused on deprived districts and areas with difficulties in access to health care provision. Finally, policy could also be directed towards entry control in terms of limiting the provision of GPs in areas with oversupply, thus favouring the undersupplied districts.

It is imperative that policy-makers introduce a system that would distribute GPs in a more equal and equitable way, taking into consideration the adjustment for inequality and the interaction of different policies, in a way that would meet the population's health needs.

## Competing interests

The author(s) declare that they have no competing interests.

## Authors' contributions

PNT and ET proposed the original idea for the study. PNT and LR collected the data. GM and PNT performed the analysis of the data. PNT, CL and GM wrote the initial manuscript. All the authors have participated in the design of the study, have commented critically on the initial manuscript and have seen and approved the final version. ET and CL are guarantors.
